# Functional analysis of *Rehmannia glutinosa* key LRR-RLKs during interaction of root exudates with *Fusarium oxysporum* reveals the roles of immune proteins in formation of replant disease

**DOI:** 10.3389/fpls.2022.1044070

**Published:** 2022-10-31

**Authors:** Chuyun Yang, Zhuomi Xie, Sheng Qian, Junyi Zhang, Zhijian Yu, Mingjie Li, Li Gu, Shuangshuang Qin, Zhongyi Zhang

**Affiliations:** ^1^ College of Agriculture, Fujian Agriculture and Forestry University, Fuzhou, China; ^2^ Key Laboratory of Ministry of Education for Genetics, Breeding and Multiple Utilization of Crops, Fujian Agriculture and Forestry University, Fuzhou, China; ^3^ College of Chemical Engineering, Huaqiao University, Xiamen, China; ^4^ Guangxi Key Laboratory of Medicinal Resources Protection and Genetic Improvement, Guangxi Botanical Garden of Medicinal Plant, Nanning, China

**Keywords:** replant disease, root exudates, Fusarium oxysporum, LRR-RLKs, immune proteins

## Abstract

Previous studies have indicated that some *Rehmannia glutinosa* Leucine-rich repeat receptor-like protein kinases (LRR-RLKs) are involved in the formation of replant disease. However, it remains unclear how the interaction of LRR-RLKs with a key factor, the interaction between root exudates and *Fusarium oxysporum*, results in formation of replant disease. In this study, the influences of root exudates, *F. oxysporum* and the interaction of these two factors on expression of nine *R. glutinosa LRR-RLKs* (*RgLRRs*) were analyzed. The resulting eight *RgLRRs* of them were highly expressed at the early stage, and rapidly declined at later stages under mixed treatment of root exudates and *F. oxysporum*. The functions of nine *RgLRRs* under root exudates, *F. oxysporum* and mixed treatment of root exudates and *F. oxysporum* were preliminarily analyzed using transient overexpression and RNAi experiments. The results showed that high expression of *RgLRR19*, *RgLRR21*, *RgLRR23* and *RgLRR29* could decrease the damage to root cells from the mixed treatment of root exudates and *F. oxysporum*, but the interference of these genes enhanced the damage levels of root cells. Based on this, stable transgenic *R. glutinosa* seedlings were acquired. Overexpression of *RgLRR29* conferred resistance of *R. glutinosa* seedlings to root exudates, *F. oxysporum* and mixed treatment. These results indicated that the continuous proliferation of *F. oxysporum* supported by root exudates altered the expression patterns of *RgLRRs* in *R. glutinosa*, then disordered the growth and development of *R. glutinosa*, finally leading to the formation of replant disease.

## Introduction

*Rehmannia glutinosa*, a plant in the Scrophulariaceae family, is a perennial herb that is widely cultivated in China (Li and Meng, 2015). *R. glutinosa* contains numerous pharmacologically active compounds, including catalpol, *Rehmannia* glycosides, *Leonurus* glycosides, *Rehmannia* polysaccharides, amino acids and stigmasterol. *R. glutinosa* is an important raw material in many traditional Chinese medicines. However, during production of *R. glutinosa*, its yield and quality are seriously affected by replant disease. Replant disease has continuously caused damage to *R. glutinosa* for at least 8-10 years (Zhang et al., 2013). Replanted *R. glutinosa* grown slowly, and the formation of tuberous roots may be arrested by replant disease (Li Q, et al., 2017). Thus, it is necessary and urgent to solve replant disease in production of *R. glutinosa*. However, effective methods to control replant disease have not been found until recently.

The majority of studies have demonstrated that an imbalance of the rhizosphere micro-ecological environment mediated by plant root exudates was the primary cause of replant disease (Grotewold and Vivanco, 2003; Xuan et al., 2005; Zhang and Lin, 2009). During the growth process of replanted plants, root exudates in the rhizosphere soils are the main source of allelotoxic substances (Bertin et al., 2003; Narula et al., 2008; Li Q et al., 2014). In previous studies on *R. glutinosa*, some secondary metabolites including ferulic acid, syringic acid and some flavonoids in root exudates, have been identified as important allelotoxic substances (Li et al., 2012; Zhang et al., 2015; Zhang et al., 2016). In addition, recent advances suggest that iridoid and phenylethanoid glycosides are also potential allelotoxic substances (Zhang et al., 2019). During the formation of replant disease, root exudates are the determining factor regulating the microbial biodiversity of the rhizosphere (Bais et al., 2006; Tu and Wu, 2010; Haichar et al., 2014; Chen et al., 2016). The abundance of *Fusarium oxysporum* spores was increased significantly in replanted *R. glutinosa* rhizosphere soils, and thus this fungus has been identified as the crucial pathogen in the formation of replant disease in *R. glutinosa* (Li et al., 2013; Li et al., 2016; Chen et al., 2019). Furthermore, root exudates of *R. glutinosa* in replanted conditions could induce proliferation of pathogenic pathogen *F. oxysporum*, resulting in aggravation of *R. glutinosa* disease (Li et al., 2016). In addition, the proliferation of *F. oxysporum* inhibited salicylic acid signal transduction and promoted the formation of replant disease (Chen et al., 2019). Therefore, current opinion holds that the interaction between root exudates and *F. oxysporum* is closely related to the formation of replant disease of *R. glutinosa* (Li et al., 2013; Wu et al., 2018). However, the interaction mechanism between root exudates and *F. oxysporum* in the formation of replant disease remains unclear.

Previous studies found that the genes related to plant immune systems were significantly upregulated in replanted *R. glutinosa* and that the interaction of these genes were closely related to the abnormal growth and death of replanted *R. glutinosa* (Chen et al., 2019; Li et al., 2017; Yang YH, et al., 2014, Yang et al., 2015). It is worth noting that in core processes of replant disease, immune system-related proteins such as Leucine-rich repeat receptor-like protein kinases (LRR-RLKs) and pathogenesis-related protein 10 (PR10) were specifically regulated in replanted *R. glutinosa* (Li MJ et al., 2017; Wu et al., 2015). The immune system of plants is primarily composed of two- layers of defense, effector-triggered immunity (ETI) and pathogen-associated molecular pattern (PAMP)-triggered immunity (PTI). The first layer, PTI, can effectively recognize PAMPs located in the cell walls using conserved regions of the genes. LRR-RLKs are a large protein family in the PTI system that play important roles in plant growth, development, and defense response (Jones and Dangl, 2006; de Lorenzo et al., 2009; Li et al., 2018). Recent research had identified 40 *RgLRRs* gene members by screening the full-length *RgLRRs* genes in the full-length transcript of *R.glutinosa*, and a total of 27 *RgLRRs* genes were found that were up-regulated in the early stages of formation of *R. glutinosa* replant disease (Xie et al., 2019). These results suggested that the immune response system, especially *RgLRRs*, may play an important role in the interaction between rhizosphere harmful microbes and allelotoxic substances. However, these *RgLRRs* genes were screened under complicated field cultivation conditions, and the expression of *R. glutinosa* genes was affected by other factors, including biotic and abiotic stressors in the soil. The key mechanism of core immunity proteins that respond to replant disease is still unknown.

In this study, the root exudates and key pathogenic microbe *F. oxysporum* were used as leading factors in simulated stresses of *R. glutinosa* under replanted conditions. The dynamic changes of pathogenic microbes in the rhizosphere soils were investigated in detail. We chose nine *RgLRRs* that were highly expressed in replanted *R. glutinosa*, and the expression and physiological index levels of the nine *RgLRRs* were examined in the roots of *R. glutinosa*. As a representative *RgLRR*, the function of *RgLRR29* under the treatments of root exudates, *F. oxysporum*, and the comprehensive stress of of root exudates and *F. oxysporum* was studied by reverse genetic methods. This study provides an effective method to examine the physiological changes and further study the expression patterns of key *RgLRRs* under replanted stress. These results will provide important clues for revealing insights into the formation mechanism of replant disease in *R. glutinosa*.

## Materials and methods

### Plant materials and stress treatments

Tissue culture seedlings of *R. glutinosa* “Wen85-5” were grown in a tissue culture room at the Institute of GAP for Chinese Medicinal Materials, Fujian Agriculture and Forestry University. Root exudates solution was acquired using a root exudates collection device constructed in previous study (Feng et al., 2022). An *F. oxysporum* strain specific to replanted *R. glutinosa* (coded No. CCS043) was prepared from five-day-old cultures in Potato Dextrose Broth (PDB) medium. *R. glutinosa* seedlings were transferred into pots (10 cm ×10 cm) filled with complex medium consisting of peat and vermiculite (v: v *=* 2:1) after acclimatization (Chen et al., 2019), and grown in a greenhouse at 28 ± 2°C with a photoperiod of 14 h: 10 h light: dark. According to previous studies, root exudates, conidial suspensions of *F. oxysporum* and a mixture of the two (root exudates and *F. oxysporum*, v: v*=* 1:1) were selected as key stress factors to treat *R. glutinosa* seedlings (Li et al., 2016; Chen et al., 2019; Feng et al., 2022). The concentration levels of root exudates were adjusted to 1.60 mg·mL^−1^ with ddH_2_O, while conidial suspensions of *F. oxysporum* were adjusted to 1×10^8^ conidia·mL^−1^. Three different solutions were used to irrigate *R. glutinosa* in pots with same volume of solution (10 mL) every two days. Each treatment was replicated three times. Root samples treated by different factors were collected at 0, 5, 10, and 15 days (DAP 0, 5, 10, and 15). Appearance of *R. glutinosa* under different treatments was carefully observed every five days. Root tips of plants from different treatments were sampled and stained by Trypan blue and DAB methods. All root samples were then cleaned and stored at −80°C after being frozen in liquid nitrogen for biochemical index and qRT-PCR analyses.

### Construction of expression vectors and definition of subcellular localization for *R. glutinosa LRR-RLKs*

Previous studies have identified 40 *R. glutinosa LRR-RLKs* from the screening of the early transcriptomic data (Xie et al., 2019). Of these, nine *RgLRRs* (*RgLRR19*, *RgLRR21*, *RgLRR23*, *RgLRR24*, *RgLRR25*, *RgLRR26*, *RgLRR27*, *RgLRR29*, and *RgLRR33*) were found to be able to specifically respond to replant disease in field (Xie et al., 2019). To construct overexpression vectors, the full-length open reading frames (ORFs) of *RgLRRs* were cloned into the entry vector pBI121-EGFP digested by *Kpn* I and *Xho* I. To build vectors for RNAi, specific fragments in the range of 100–300 bp in the ORFs of *RgLRRs* were amplified. The specific fragments were cloned into the entry vector pRNAiGG (Yan et al., 2012).

For subcellular localization of the nine *RgLRRs*, the *RgLRRs* were fused with EGFP. The EGFP fusion constructs were driven by the double 35S promoter. All primers used in vector construction are shown in **Tables S1a–c**. *Agrobacterium tumefaciens* strain GV3101 containing 35S::RgLRRs-EGFP and 35S::GFP (used as a control) were grown overnight in LB solution containing 50 μg·mL^−1^ Kan and 100 μg·mL^−1^ Rif, and then adjusted to OD600 = 0.8. The bacterial solution was resuspended with the injection buffer (10 mM MgCl_2_, 100 mM 2-morpholinoethanesulfonic acid, and 200 μM acetosyringone (AS), pH = 5.8) and injected into leaves of *Nicotiana benthamiana* using a needle syringe. At 48 h after injection, the EGFP signals were observed with a laser scanning confocal microscope (LEICA TCS SP8).

### Quantification of *F. oxysporum*

The rhizosphere microbial DNA was extracted using a reference extraction kit (Beijing Tianmo, TD601, China). The quantity of *F. oxysporum* was detected using absolute quantification PCR (Chen et al., 2019). The specific primers of *F. oxysporum* (ITS1-F: 5’-CTTGGTCATTTAGAGGAAGTAA-3’, ITS4-R: 5’-TCCTCCGCTTATTGATATGC-3’) were used to amplify the DNA fragments using touchdown PCR. The bright electrophoretic strips of target genes were extracted using a gel pure DNA Kit (Magen, Guangzhou, China), and were inserted into a pMD19-T vector. Then, the vector DNA solution was transformed into *E. coli* DH 5α. One or two single white colonies were selected to identify whether the target genes were correctly cloned into the vector. The solutions containing the appropriate size of DNA fragments were chosen to extract plasmids using a Hipure Plasmid Micro Kit (Magen, Guangzhou, China) following the manufacturer’s instructions.

Plasmid solutions containing DNA fragments of correct size were amplified by qRT-PCR using the plasmid primer RV-M/M13-47. The concentration of target gene DNA was measured by a Nanodrop2000 spectrophotometer (Thermo Scientific, USA) and diluted to 0,1, 2, 3, and 4 ng·μL^–1^. The standard curve was drawn according to the DNA concentration of the target gene and Ct value. Finally, the soil DNA extracts were detected by qRT-PCR using *F. oxysporum* specific primers. The copy number of *F. oxysporum* spores were calculated from the standard curve. Each gene was analyzed with three replicates.

### Chemical tissue staining

The root samples were used to detect the presence of *in situ* accumulation of superoxide and H_2_O_2_ by staining with 3,3-diaminobenzidine (DAB) and Trypan blue, respectively. In brief, root tips 2.0–2.5 cm in length were immediately immersed in an aqueous solution of 1 mg·mL^–1^ DAB in 50 mmol·L^–1^ potassium phosphate buffer (pH=6.4) and vacuum infiltrated and incubated for 12 h in the dark according to a previous report (Zhang et al., 2017). The root tips were placed in an ethanol: lactic acid: glycerol (3:1:1) mixture to boil for 5 min, and stored in 60% glycerol. The root tips were washed three times with water before photographing 10–15 individuals randomly sampled from each group in each experiment. According to the method of Liu (Liu et al., 2016), the root tips were immersed in 10% KOH, at 90°C for 1 h; the KOH solution was discarded, and the root tips were washed with ddH_2_O. Then, 1 mL of 2% HCl solution was added in the centrifuge tubes allowed to stand for 90 min. The HCl solution was discarded, and 1 mL Trypan blue staining solution was added (the final concentration of Trypan blue was 10 mg·mL^-1^) and stained for 30 min. The root tips were removed and added to 1 mL ethanol for overnight decolorization before photography using fluorescence microscope (Leica DM5000 B, Leica Microsystems Ltd., Heerbrugg, Switzerland).

### Measurement of antioxidant enzyme activities and detection of malondialdehyde and chlorophyll content

The root samples of *R. glutinosa* seedlings under root exudates, *F. oxysporum* and the mixed solution of root exudates and *F. oxysporum* were used in these assays. Approximately 2 g of root samples were homogenized in 3 mL of 50 mM potassium phosphate buffer (pH=7.0). The supernatant was collected by centrifugation at 10,000×g for 10 min at 4°C, and then used for the activity determination of peroxidase (POD), catalase (CAT), superoxide dismutase (SOD), and malondialdehyde (MDA). POD activity was determined as guaiacol oxidation by H_2_O_2_. SOD activity was analyzed based on the inhibition rates of the reduction of nitro blue tetrazolium (NBT), CAT activity was determined as the H_2_O_2_ consumption (Aebi, 1983). MDA content was determined by the thiobarbituric acid reaction method according to our previous report (Peng et al., 2019). A chlorophyll meter (SPAD-502, Minolta Camera Co. Japan) was used to determine the total chlorophyll content according to our previous study (Wang et al., 2021).

### Measurement of salicylic acid content

The content of salicylic acid (SA) was detected using a one-step double-antibody sandwich enzyme-linked immunosorbent assay (ELISA). The content of SA was measured at 450 nm using a microplate reader (BIO-Tek ELX800, USA). Calculations of the ELISA data were performed as described in Wang et al. (2012).

### Transient overexpression and RNAi of *R. glutinosa LRR-RLKs*

The overexpression and RNAi vectors of *RgLRRs* were constructed in this study. The resulting constructs were transformed with the *Agrobacterium tumefaciens* GV3101 strain using the freeze-thaw method and verified by sequencing (Sangon, Shanghai, China). The *A. tumefaciens* strains GV3101 harboring *RgLRRs*-overexpression (*RgLRRs*-OX) or *RgLRRs*-RNAi (*RgLRRs*-Ri) transformation constructs were grown overnight in LB culture solution. The bacteria solutions were adjusted to OD600 = 0.8 and set to 5000/rpm for 5 min, and the bacteria were resuspended with the infection solution (containing 100 μM AS). To transiently transform *RgLRRs* genes in isolated tuber roots of *R. glutinosa*, 1 cm diameter segments of root tubers of *R. glutinosa* were selected and washed using flowing water for 1 h, and then soaked in 75% alcohol for 60 s. The root segments were then cut into 2 mm segments with the same thickness and equal size with a sterile scalpel, and treated in 0.3 MPa for 3 h with different infection solutions using a vacuum pump, respectively. The infected root segments were dried and connected to Murashige and Skoog (MS) solid medium containing 50 mg·L^–1^ AS. After dark culture for two days, these root segments were transferred into 1% agarose plate that were pre-cultivated in Petri dish at 26°C. Then, 20 μL root exudates solution, 20 μL conidial suspension of *F. oxysporum*, or 20 μL mixed solution of root exudates and *F. oxysporum* were added to the root segments every 12 h. Simultaneously, the area of mycelial expansion was used to test the resistance of root segments, the antioxidant enzyme activities, MDA content, and gene expression levels were determined for different treatments of root segments.

### Establishment and confirmation of *R. glutinosa* transformation

The overexpression vector of *RgLRR29* was constructed in this study. The full-length ORF of *RgLRR29* was cloned into pBI121-EGFP digested by *Kpn* I and *Xho* I sites. The expression of *RgLRR29* was driven by double 35S promoters. For *R. glutinosa* seedling regeneration, approximately 20-day-old leaves were detached from the *R. glutinosa* seedlings, and cut along straight lines. The 100 leaf explants were dipped into a bacterial suspension (OD600 = 0.8) of *A. tumefaciens* GV3101, harboring the *RgLRR29* transformation construct. After 30 min, the leaf explants were blotted dry with autoclaved filter paper, placed in MS basal medium containing 100 μM AS, and cultured at 25°C in the dark for two days. Then the leaf explants were transferred into differentiation medium containing 0.5 mg·L^-1^ NAA and 2 mg·L^-1^ 6-BA under a 12-h light/12-h dark photoperiod for seedling regeneration, with the medium replaced with fresh medium every 15 days until seedling regeneration.

To evaluate whether the overexpressed *RgLRR29* had been integrated into the transgenic *R. glutinosa* genome, total genomic DNA was isolated from the leaves of the transgenic lines using the cetyltrimethylammonium bromide (CTAB) method. DNA of transgenic plants was detected through the *Kan* gene with specific primers (*Kan*-F: CGTTCCATAAATTCCCCTCG; *Kan*-R: ATCTCGTGATGGCAGGTTGG). The expression level of *RgLRR29* was determined by qRT-PCR.

### Roles of *RgLRR29* during interaction between root exudates and *F. oxysporum*

The *RgLRR29-*OX lines were used to assess the expression levels by qRT-PCR. Then, the *RgLRR29-*OX and wild-type (WT) seedlings were transplanted into pots (20 cm×18 cm) containing organic matrix nutrition soils and grown in a greenhouse under 25°C with a photoperiod of 14 h: 10 h light: dark until the roots of the *R. glutinosa* expanded to the harvest period. The tuberous roots of *RgLRR29-*OX and WT *R. glutinosa* plants were used to culture the next generation of plants. When the fibrous roots were developed and elongated, then the tuberous roots were removed from the plants for avoiding influence of other factors. For studying the change of phenotype and physiological response in replanted *R. glutinosa*, *RgLRR29-*OX and WT seedlings were planted in plastic pots (10 cm ×10 cm), root exudates solution, conidial suspension of *F. oxysporum* and the mixed solution of root exudates and *F. oxysporum* spores were added, and the seedlings were grown in the above-mentioned greenhouse under normal field management for fifteen days. The roots of *RgLRR29-*OX and WT seedlings were sampled, immediately frozen in liquid nitrogen, and stored at –80°C for further analyses regarding gene expressions and physiological indexes.

### Gene expression analysis

Total RNA was isolated from 100 mg of *R. glutinosa* roots (fresh-weight) using a plant RNA extraction kit (Nanjing Vazyme Biotech Co., Ltd.). The cDNA synthesis was performed with the Evo M-MLV Mix Kit with gDNA Clean for qRT-PCR AG11728 (Accurate biotechnology (Hunan) Co., Ltd.). Each reaction contained 10 µL of 2 × SYBR Green Pro Taq HS Premix AG11701 (Accurate Biotechnology (Hunan) Co., Ltd.), 2 µL of template cDNA and 0.4 µL of each forward and reverse primers (10 µM). The data were normalized on the basis of the 18S rRNA (DQ469606) threshold cycle (Ct) value. All primers used in this experiment are shown in **Table S1d**. The qRT-PCR reaction procedure was performed as follows, incubation at 95°C for 2 min followed by 40 cycles at 95°C for 5 s and 60°C for 30 s. Each gene was tested in triplicates with three technical repeats. The expression level for each sample was expressed by the 2^−△△CT^ method (Livak and Schmittgen, 2001). The data were exhibited as the mean ± SD of three independent experiments.

### Statistical analysis

Raw data were compiled and regression analyses and graphs were prepared using Microsoft Excel. Multiple comparisons (LSD) were used to evaluate the significant differences between the compared values. Each value with three replicates represented as the mean ± SD. p < 0.05 was considered as significant between any two groups.

## Results

### Changes of phenotypic characteristics in *R. glutinosa* under treatment with key factors leading to the formation of replant disease

**Figure 1:**
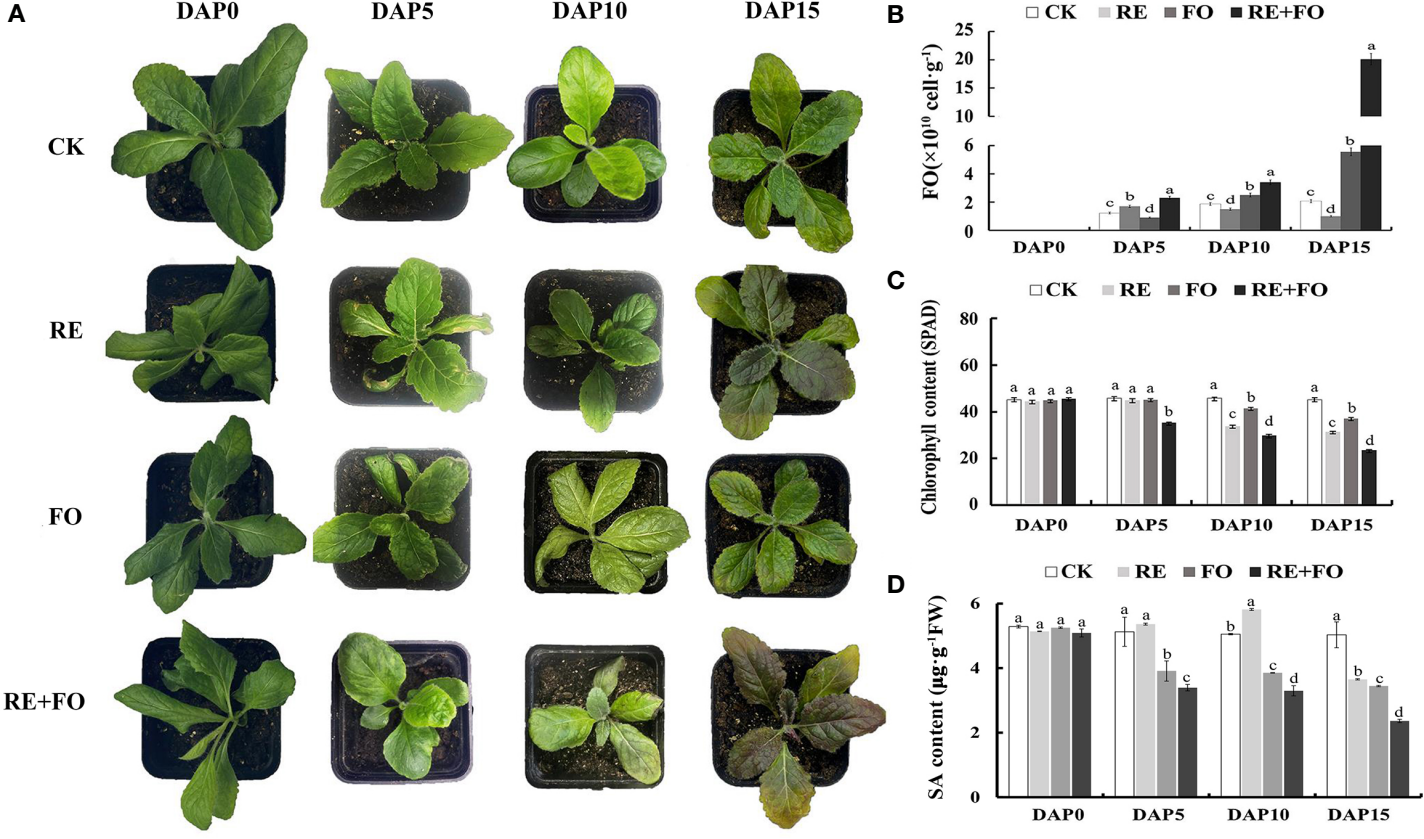
Morphological differences, *Fusarium oxysporum* proliferation, chlorophyll content and Salicylic acid (SA) content analysis of *Rehmannia glutinosa* seedlings under root exudates, *F*. *oxysporum* and comprehensive treatment of root exudates and *F*. *oxysporum*. **(A)** Phenotypic changes of *R. glutinosa* seedlings under root exudates, *F*. *oxysporum* and comprehensive treatment of root exudates and *F*. *oxysporum*. There were three biological replicates for each treatment; **(B)** Analysis of *F.oxysporum* proliferation in *R. glutinosa* rhizosphere soils under root exudates, *F*. *oxysporum* and comprehensive treatment of root exudates and *F*. *oxysporum*; **(C)** Chlorophyll content analysis of *R. glutinosa* under root exudates, *F*. *oxysporum* and comprehensive treatment of root exudates and *F*. *oxysporum*. **(D)** SA content analysis of *R. glutinosa* under root exudates, *F*. *oxysporum* and comprehensive treatment of root exudates and *F*. *oxysporum*. DAP, Days after planting; CK, Normalplanting “Wen85-5” R; glutinosa; RE, the treatment of root exudates; FO, the treatment of F. oxysporum; RE+FO; the comprehensive treatment of root exudates and *F*. *oxysporum*. Different letters represent a significant difference at *P* < 0.05.

In previous study, a specific culture device was designed to isolate high-purity of root exudates of *R. glutinosa* (Feng et al., 2022). In this study, root exudates of *R. glutinosa* were collected based on this device. The root regions of *R. glutinosa* seedlings were irrigated with root exudates solution, conidial suspension of *F. oxysporum* and the mixed solution of root exudates and *F. oxysporum* spores to further investigate the interaction between root exudates and *F. oxysporum*. At 10 days after treatments, the copy number of *F. oxysporum* in the comprehensive treatment of root exudates and *F. oxysporum* was 1.82, 2.27 and 1.36 times of those in the control (CK), single root exudates and *F. oxysporum* treatments, respectively. At 15 days after treatments, the copy number of *F. oxysporum* in the comprehensive treatment of root exudates and *F. oxysporum* was 3.62 times of that in the *F. oxysporum* treatment group (**Figure 1B**). Appearance and key physiological indicators of *R. glutinosa* seedlings were analyzed to explore the effects of different treatments on the growth of *R. glutinosa*. Leaves of *R. glutinosa* treated by the comprehensive stress of root exudates and *F. oxysporum* displayed deeper yellowing and serious wilting, and some leaves presented symptoms of purple leaves in comparison to the controls (**Figure 1A**). At 15 days after treatment, the chlorophyll contents of leaves in the comprehensive treatment of root exudates and *F. oxysporum* were significantly lower than in the root exudates and *F. oxysporum* groups. The chlorophyll content for *R. glutinosa* leaves under both root exudates and *F. oxysporum* treatment decreased gradually compared with controls, but the chlorophyll content of the comprehensive treatment of root exudates and *F. oxysporum* was the lowest (**Figure 1C**). In the process of three treatments, the content of SA in the roots of plants under the treatment of *F. oxysporum* and the comprehensive treatment of root exudates and *F. oxysporum* gradually decreased, and the content of SA under root exudates treatment was firstly increased but then decreased, and the content of SA under the comprehensive treatment of root exudates and *F. oxysporum* was the lowest (**Figure 1D**). The results were consistent with previous studies in *R. glutinosa* treated by single or a combination of representative allelotoxic substances (Li et al., 2016; Feng et al., 2022), again indicating that root exudates was the key factor promoting the proliferation of *F. oxysporum* in the *R. glutinosa* rhizosphere.

### Changes of antioxidant enzyme activities and oxidative damage in *R. glutinosa* roots mediated by key replant disease formation factors

**Figure 2:**
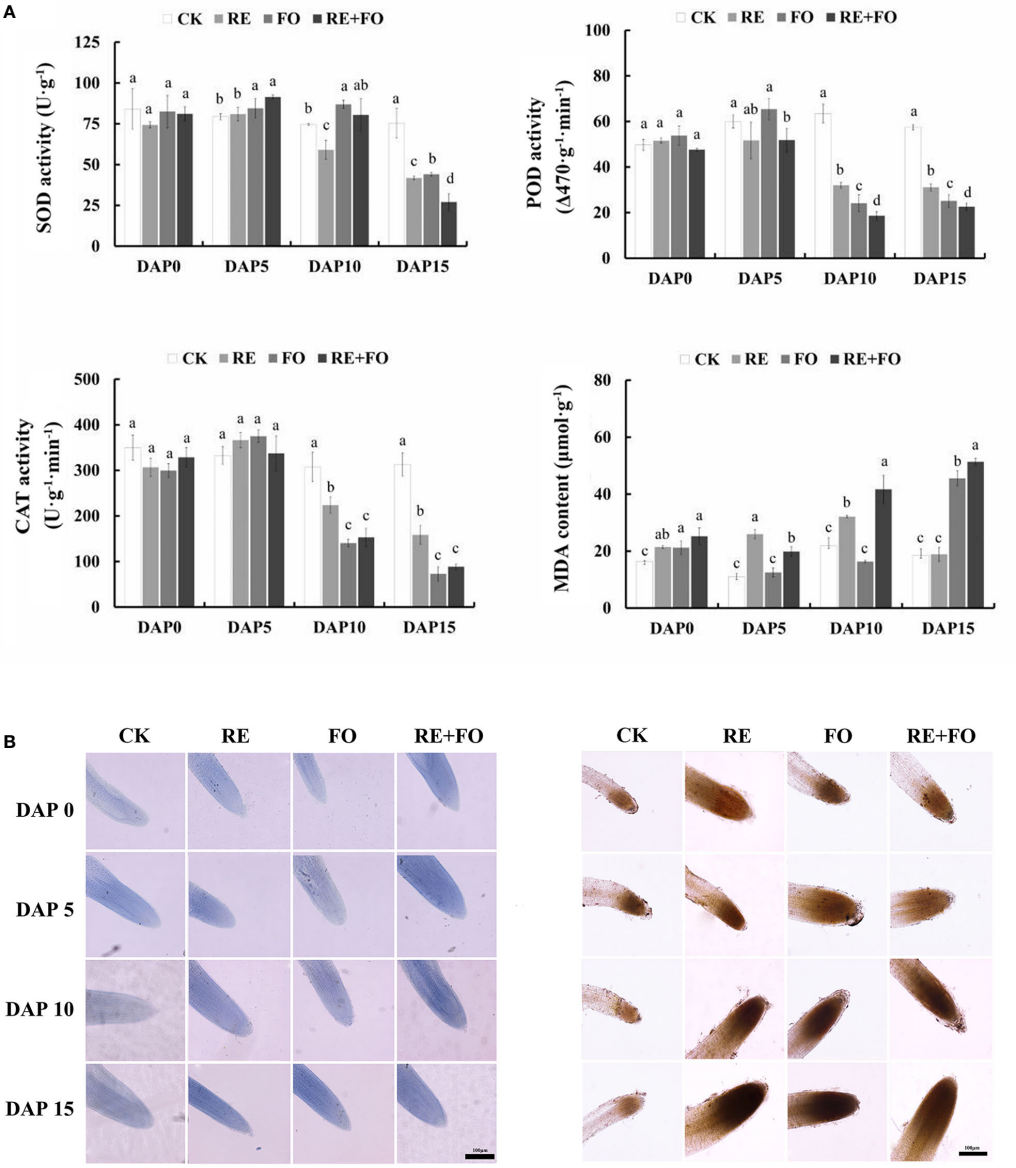
Changes in physiological indexes **(A)** and reactive oxygen species (ROS) levels **(B)** of *Rehmannia glutinosa* seedlings under root exudates, *F*. *oxysporum* and comprehensive stress of root exudates and *F*. *oxysporum*. **(A)**: Measurement of SOD, POD, CAT and detection of MDA in *R. glutinosa* roots under root exudates, *F*. *oxysporum* and comprehensive stress of root exudates and *F*. *oxysporum*; **(B)**: ROS levels of *Rehmannia glutinosa* root tip stained with Trypan blue (left) and DAB (right) mediated by the treatments of root exudates, *F*. *oxysporum* and comprehensive stress of root exudates and *F*. *oxysporum*. Bar = 100 μm. DAP, Days after planting; RE, the treatment of root exudates; FO, the treatment of F.oxysporum; RE+FO, the comprehensive treatment of root exudates and F. oxysporum. Different letters represent a significant difference at *p* < 0.05.

The antioxidant enzyme activities of *R. glutinosa* seedlings roots were determined to study the damage level of *R. glutinosa* seedlings in treatments of root exudates, *F. oxysporum* and comprehensive stress of root exudates and *F. oxysporum*. The trends of POD and CAT activities were similar to SOD activities showing an upward trend in the early treated stages (days 1–5) and a downward trend at days 5–15 in comparison to controls (**Figure 2A**). Simultaneously, the MDA content of *R. glutinosa* seedlings under different treatments increased gradually with increasing treatment time under each treatment. Overall, the comprehensive treatment of root exudates and *F. oxysporum* significantly promoted the accumulation of reactive oxygen species (ROS) in *R. glutinosa* seedlings compared to treatment with single root exudates or *F. oxysporum*.

To further determine the oxidative damage of *R. glutinosa* seedlings under root exudates, *F. oxysporum* and comprehensive stress of the two factors visually, the oxidative damages levels of plant cells in *R. glutinosa* roots were detected by Trypan blue and DAB staining. The results suggested that the levels of damage of the root cells in *R. glutinosa* treated with root exudates, *F. oxysporum* and comprehensive stress of the two factors gradually deepened with increasing of treatment time compared to controls (**Figure 2B**). The reactive oxygen species content could be detected by the color depth in the root tips of *R. glutinosa*. It is worth noting that the root tips of plants treated with comprehensive stress of root exudates and *F. oxysporum* were the most severely damaged compared with the controls, root exudates or *F. oxysporum* treatments. These results indicated that the comprehensive treatment of root exudates and *F. oxysporum* increased the damage levels of *F. oxysporum* to *R. glutinosa*.

### The subcellular localization of *R. glutinosa* LRR-RLK proteins

**Figure 3:**
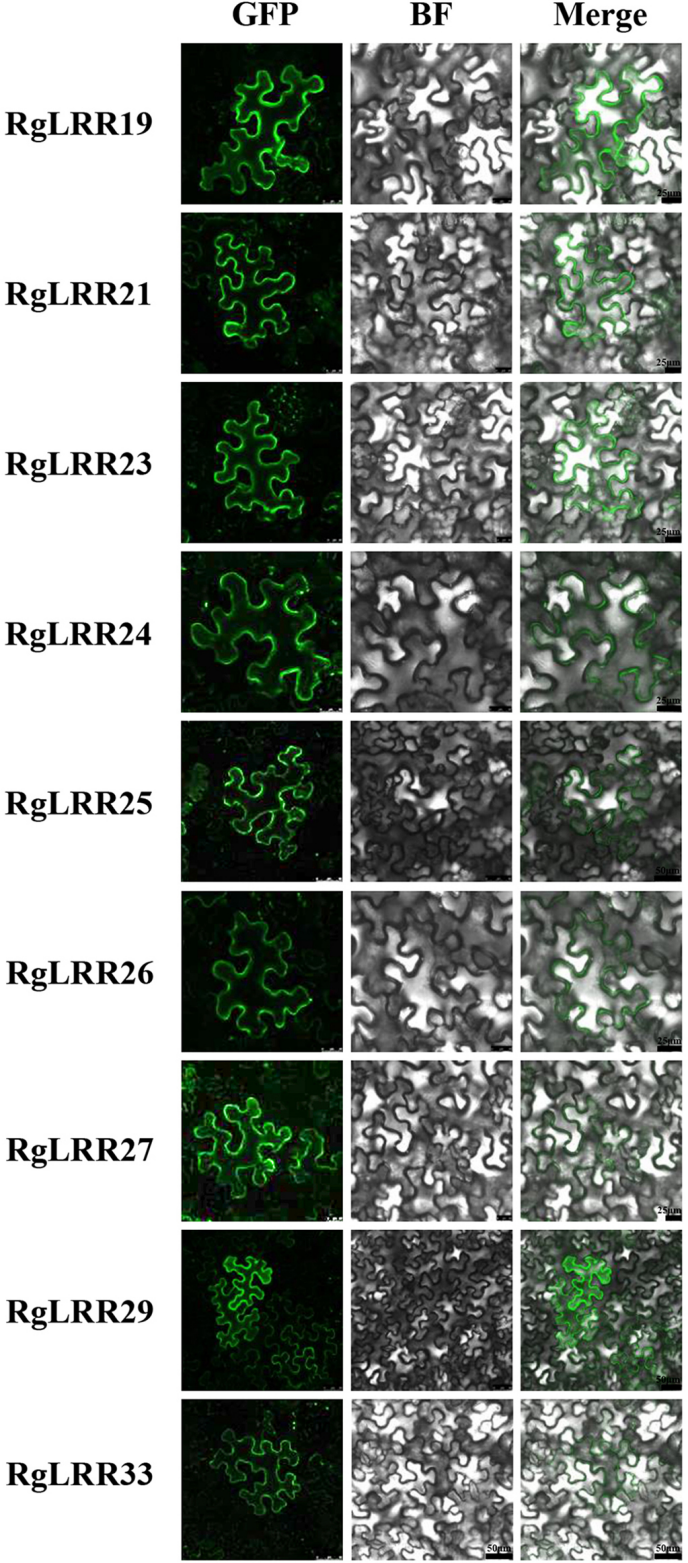
Subcellular localization of RgLRRs in *Nicotiana benthamiana*. GFP, Green fluorescence protein; BF, Bright field. There were five replicates for each experiment.

To reveal the molecular functions of these *RgLRR* genes, the nine *RgLRRs* was further cloned from *R. glutinosa* roots. To determine the localization of RgLRRs in cells, the complete coding regions of the nine RgLRRs were fused to the N-terminus of EGFP. The 35S::GFP-RgLRRs constructs were transiently expressed in leaves of *N. benthamiana*, and the green fluorescence from fusion proteins of 35S::GFP-RgLRRs was detected by fluorescence microscopy. The resulting green fluorescence were clearly observed in the cell membranes of *N. benthamiana* leaves (**Figure 3**). The results indicated that these RgLRRs of *R. glutinosa* were all located in the cellular membranes, similar to the cellular position of most plant LRR-RLK proteins.

### Expression patterns of *RgLRRs* Genes in *R. glutinosa* roots mediated by key replant disease formation factors

**Figure 4:**
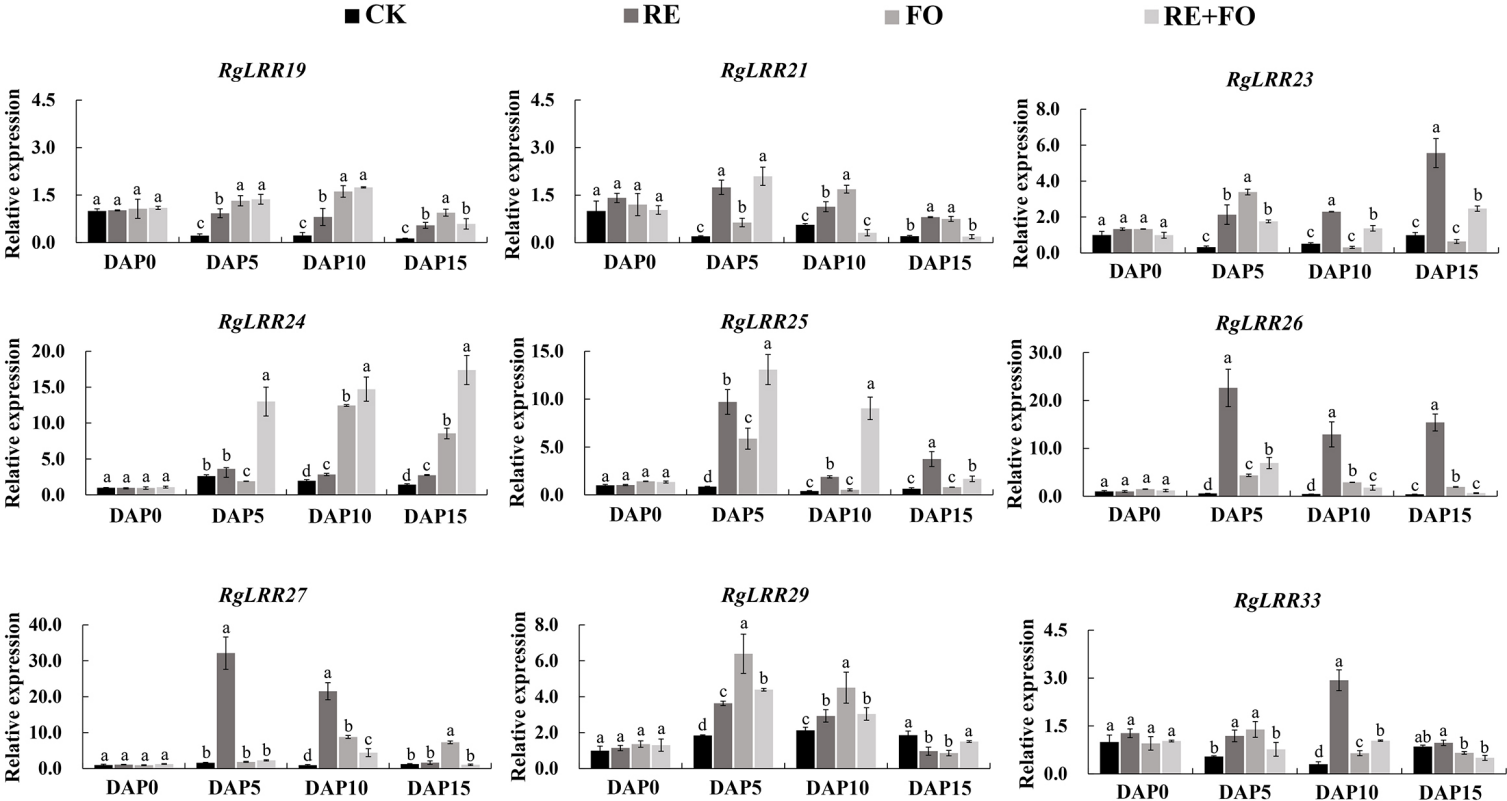
Expression patterns of crucial *RgLRRs* in *Rehmannia glutinosa* under the treatments of root exudates, *F. oxysporum* and comprehensive stress of root exudates and *F. oxysporum*. DAP, Days after planting; RE, the treatment of root exudates; FO, the treatment of F. oxysporum; RE+FO,the comprehensive treatment of root exudates and F. oxysporum. Different letters represent a significant difference at *p* < 0.05.

To examine the effects of root exudates, *F. oxysporum*, and their interaction in *R. glutinosa*, the expression patterns of *RgLRRs* in *R. glutinosa* under the treatments of root exudates, *F. oxysporum* and comprehensive stress of them were analyzed by qRT-PCR. The results showed that the expression patterns of the nine *RgLRRs* could be divided into three groups according to their expression patterns (**Figure 4**). The first group included *RgLRR33* was weakly significantly expressed during the treatments. The second group consisted of *RgLRR19*, *RgLRR21*, *RgLRR25*, *RgLRR26*, *RgLRR27*, and *RgLRR29*, that were highly expressed at early stages and finally down-regulated at 15 days after three treatments. In the third group, the expression levels of *RgLRR23* and *RgLRR24* grew steadily. The expression trends of *RgLRRs* were consistent in that they were up-regulated at first but decreased gradually with increasing of *F. oxysporum* treatment time. However, *RgLRR21*, *RgLRR23*, *RgLRR26*, *RgLRR29*, and *RgLRR33* were down-regulated at 10 days after the treatment of *F. oxysporum*, and other *RgLRRs* were down-regulated at 15 days after the treatment of *F. oxysporum*. From the expression profiling of all three groups, except for *RgLRR33*, the other eight *RgLRRs* all responded significantly to the treatment of comprehensive stress of root exudates and *F. oxysporum*.

### Changes of antioxidant enzyme activities and MDA content of *R. glutinosa* segments under the treatments of root exudates, *F. oxysporum* and their interaction after transient overexpression and RNAi of key *RgLRRs*

**Figure 5:**
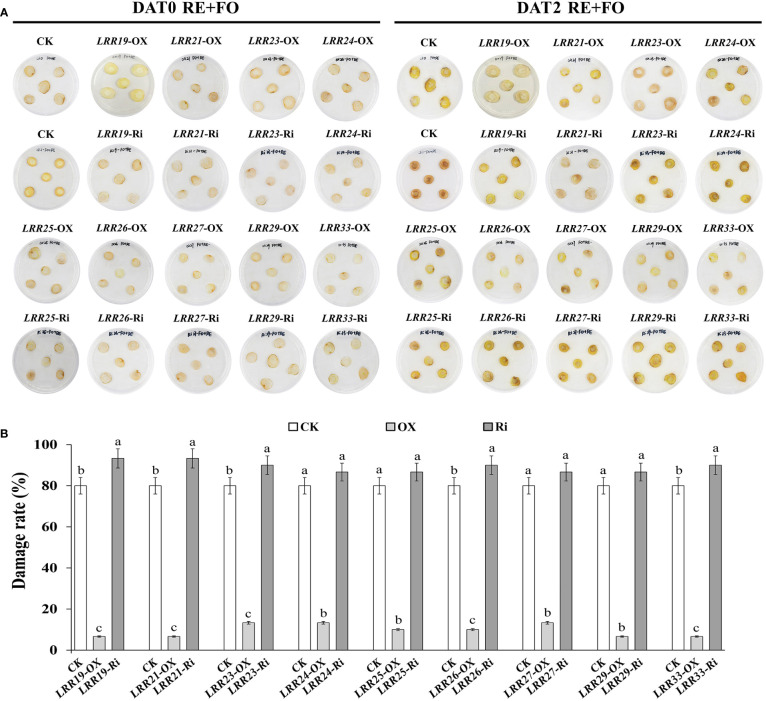
Phenotypic changes **(A)** and damage rate **(B)** of root segments with transient overexpression and interference of different *RgLRRs* under the treatment of comprehensive stress of root exudates and *F. oxysporum*. OX, Overexpression; Ri, RNA interference; CK, Empty vector as control; DAT, Days after treatment; RE+FO, the comprehensive treatment of root exudates and *F. oxysporum*. Different letters above bars indicated significant differences at P = 0.05 using Student-Newman-Keuls multiple comparisons.

To preliminarily identify the functions of *RgLRRs* in different key replant disease factor stresses, nine *RgLRRs* (*RgLRR19*, *RgLRR21*, *RgLRR23*, *RgLRR24*, *RgLRR25*, *RgLRR26*, *RgLRR27*, *RgLRR29* and *RgLRR33*) were transiently transformed into root segments and further treated by root exudates, *F. oxysporum* and comprehensive stress of them. The phenotypic changes from the transient *RgLRRs* overexpression and RNAi in *R. glutinosa* root segments under the treatments of root exudates, *F. oxysporum* and comprehensive stress of them were further observed (**Figure S1**). The results showed that the transient overexpression of *RgLRRs* genes in root segments of *R. glutinosa* except *RgLRR33* showed higher resistance to these three treatments, but transient RNAi of *RgLRRs* genes resulted in the root segments (**Figure 5A**), which suffering increased damage, indicating that the transient overexpression of *RgLRRs* genes had an effect on the resistance to these stresses. In addition, after transient overexpression of different *RgLRRs* genes, the root segments showed different levels of resistance to the treatments of root exudates, *F. oxysporum* and comprehensive stress of them. The transient overexpression of *RgLRR26* and *RgLRR33* was associated with higher resistance to the treatment of root exudates. However, *RgLRR19*, *RgLRR25*, *RgLRR26* and *RgLRR29* may also play positive roles in the resistance to the effects to the treatment of *F. oxysporum*. In addition, transient overexpression of *RgLRR19*, *RgLRR21*, *RgLRR23* and *RgLRR29* resulted in the lowest degree of damage under the comprehensive treatment of root exudates and *F. oxysporum* (**Figure 5B**).

**Figure 6:**
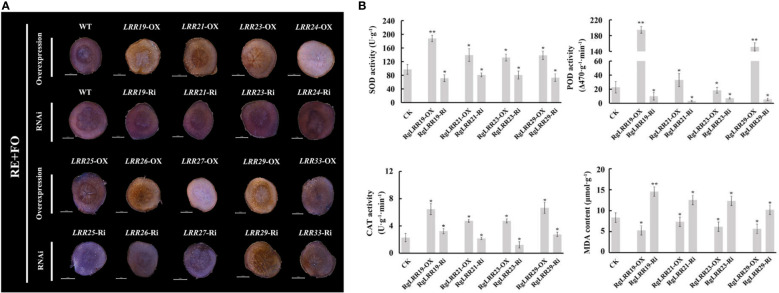
Changes of root segments with transient overexpression and RNAi of nine *RgLRRs* under comprehensive treatment of root exudates and *F*. *oxysporum* **(A)**, Trypan blue staining of root segments with transient overexpression and RNAi of nine *RgLRRs* under comprehensive treatment of root exudates and *F*. *oxysporum*; **(B)**, The physiological indexes analysis of root segments with transient overexpression and RNAi of crucial *RgLRRs* under comprehensive treatment of root exudates and *F*. *oxysporum*. OX, Overexpression; Ri, RNA interference; CK, Empty vector as control; RE+FO, the comprehensive treatment of root exudates and *F*. *oxysporum*. Asterisks indicate significant difference compared with the corresponding controls (*, P < 0.05; **, P < 0.01).

To further explore the effects of these treatments on *R. glutinosa* root segments with transiently overexpressed and interfered *RgLRRs*, the antioxidant enzyme activities and MDA content were analyzed. As a result, the activities of SOD, POD and CAT in root segments with transient overexpressed *RgLRRs* were higher than those treated with empty vectors in *R. glutinosa* root segments, with lower content of MDA in root segments with transient overexpression of *RgLRRs*. It can be seen that *RgLRR19*-OX, *RgLRR21*-OX, *RgLRR23*-OX and *RgLRR29*-OX had the lowest damage degree of hydrogen peroxide through trypan blue staining results of root slices (**Figure 6A**). The opposite results occurred after RNAi of *RgLRRs* (**Figures S1**, **S2**). It was worth noting that under the comprehensive treatment of root exudates and *F. oxysporum*, the antioxidant enzyme activities of root segments with transient overexpressed *RgLRR19*, *RgLRR21*, *RgLRR23* and *RgLRR29* were higher than those of controls, and the MDA contents were lower (**Figure 6B**). These results indicated that the expression levels of *RgLRRs* may be related to resistance of the root cells to root exudates and *F. oxysporum*.

### The overexpression of *RgLRR29* in *R. glutinosa* significantly affected the resistance of *R. glutinosa* against replant disease

**Figure 7:**
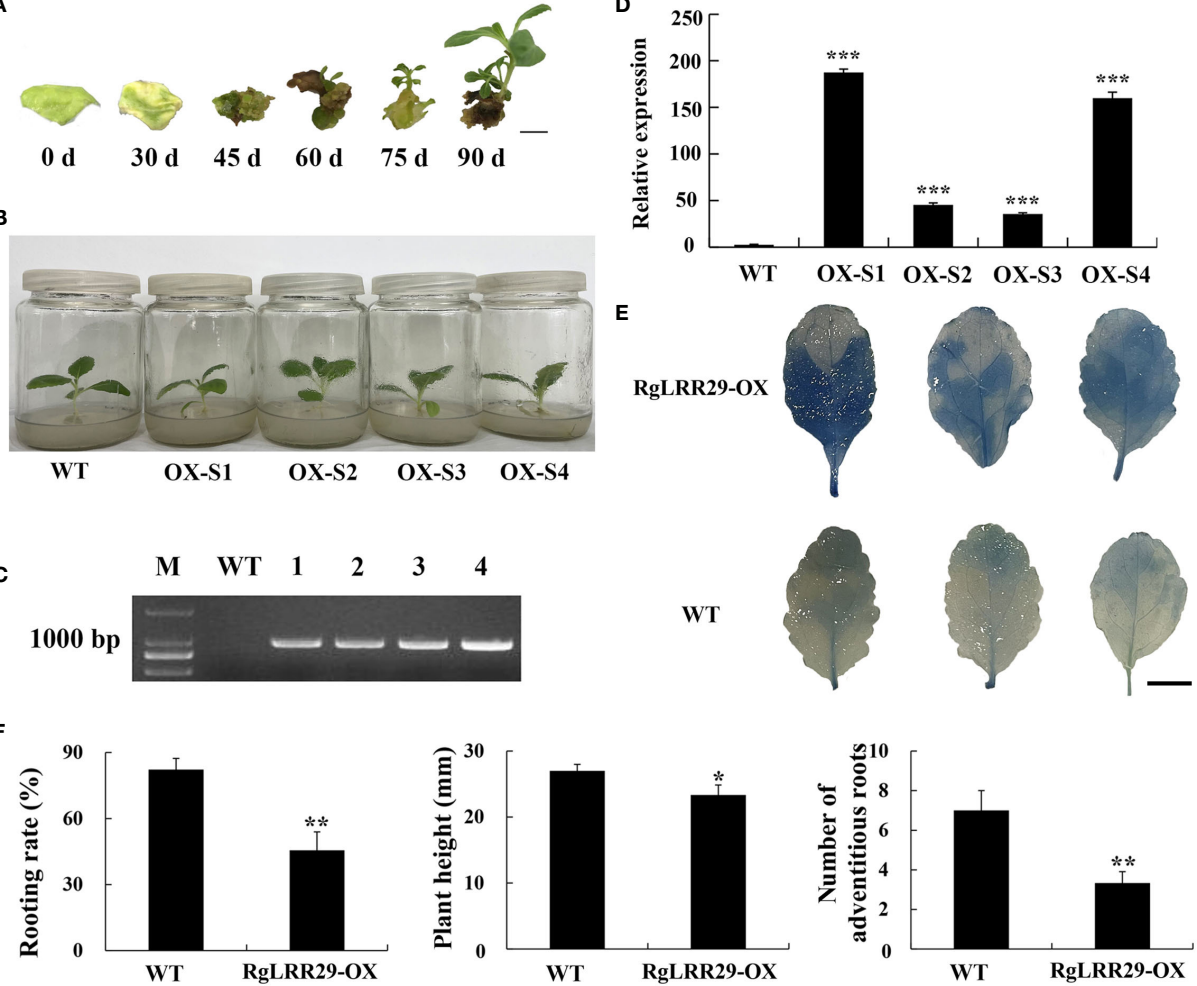
Confirmation of positive *RgLRR29*-overpression *Rehmannia glutinosa*. **(A)** Regeneration process of *RgLRR29*-overexpression *R. glutinosa* plants. Bar = 1cm; **(B)** Morphology of *RgLRR29*-overexpression *R. glutinosa* plants cultured in MS medium for three weeks. OX-S1, OX-S2, OX-S3, and OX-S4 represent different lines of *RgLRR29*-overexpression *R. glutinosa* plants. Wild-type (WT) plants of the same age are shown as the control; **(C)** PCR products for the positive screening of transgenic lines; Lanes M and WT represented the DL2000 size marker and WT plants, respectively; Lanes 1–4 represented *RgLRR29*-overexpression lines 1 to 4, respectively; **(D)** Expression patterns of different *RgLRR29*-overexpression lines (OX-S1, OX-S2, OX-S3, OX-S4) in roots. The error bars represent the standard error (n = 3) (***: *p* < 0.001); **(E)** ROS accumulation in *RgLRR29*-overexpression and WT *R. glutinosa* plants. Trypan blue staining was used to detect ROS accumulation in the leaves of 2-week-old *RgLRR29*-overexpression and WT *R. glutinosa* plants; **(F)** The rooting rate, Plant height and the number of adventitious roots of WT and *RgLRR29*-overexpression *R. glutinosa* plants. The rooting rate was calculated from three independent experiments. More than 15 plants were subjected to root induction each time. Other data are shown for one representative result of three independent experiments; the results are shown as the average ± SE (n = 5). *P < 0.05 and **P < 0.01 indicate significant differences based on the t-test.

As a representative *RgLRR*, in this experiment, *RgLRR29* was overexpressed in tissue-cultured *R. glutinosa* seedlings *via Agrobacterium*-mediated transformation using the leaf disc method. Leaf explant of *R. glutinosa* infected by *Agrobacterium* were regenerated on plates with resistance. The resulting 30-day shoots infected by *Agrobacterium* with *RgLRR29* with resistance emerged from leaf explants. These shoots were grown to form complete culture seedling of *R. glutinosa* with over expressed *RgLRR29* (*RgLRR29*-OX) for 90 days (**Figure 7A**). Then after three weeks, the root morphogenesis of WT and transgenic *R. glutinosa* shoots cultured in rooting medium was established (**Figure 7B**). After these candidate transgenic *RgLRR29*-OX lines were fully acclimatized, they were transferred into pots with peat and vermiculite matrix. Simultaneously, PCR was used to validate the positive *RgLRR29*-OX lines in these new *R. glutinosa* seedling that emerged from the tuberous roots. The results showed that 952 bp of the *kanamycin* gene were specifically amplified from the DNA of 4 transgenic plants (*RgLRR29*-OX-S1, -S2, -S3 and -S4) while the WT (wild-type) seedlings showed no amplification (**Figure 7C**). Simultaneously, qRT-PCR was used to analyze the expression levels of *RgLRR29* in *RgLRR29*-OX lines and wild lines. Compared with the WT, the expression levels of the *RgLRR29* in OX-S1#, OX-S2#, OX-S3#, and OX-S4# increased by 75, 18, 14, and 64-fold, respectively. Resulting the *RgLRR29*-OX-S1 and *RgLRR29*-OX-S4 lines presented higher expression levels of *RgLRR29* compared with WT seedlings (**Figure 7D**). ROS accumulation was significantly induced in *RgLRR29-*OX leaves compared with WT plant leaves of the same age (**Figure 7E**). In this study, we found that plant height, the number of adventitious roots and the rooting rate of *RgLRR29*-overexpression plants were significantly lower than those of WT plants when they were cultured in MS medium (**Figure 7F**). These results indicated that transgenic *R. glutinosa* seedlings of overexpression *RgLRR29* were successfully acquired, thereby providing materials for verifying its function in the treatments of root exudates, *F. oxysporum* and comprehensive stress of root exudates and *F. oxysporum*.

**Figure 8:**
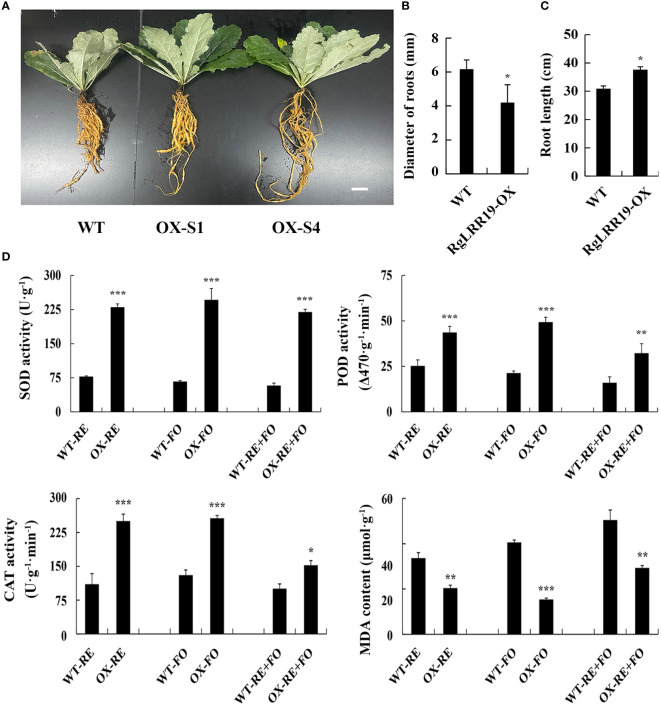
*RgLRR29-*overexpression improves plant resistance against root exudates, *F. oxysporum* and comprehensive stress of root exudates and *F. oxysporum.* **(A–C)**, Phenotypic analysis of the WT and transgenic lines. Bar = 2 cm. **(D)** Changes of physiological indexes in WT and *RgLRR29*-overexpression *Rehmannia glutinosa* plants under the treatments of root exudates, *F. oxysporum* and comprehensive stress of root exudates and *F. oxysporum*. RE, the treatment of root exudates; FO, the treatment of *F. oxysporum*; RE+FO, the comprehensive treatment of root exudates and *F. oxysporum*. The error bars represent the standard error (n = 3) (*: *p* < 0.05; **: *p* < 0.01; ***: *p* < 0.001).

After 120 days, *RgLRR29*-OX-S1, *RgLRR29*-OX-S4, and WT *R. glutinosa* seedlings generated tuberous roots (**Figure 8A**). The diameter of roots of *RgLRR29*-overexpressing plants was less than WT plants (**Figure 8B**). However, the root length of *RgLRR29*-overexpressing plants was longer than WT plants (**Figure 8C**). To further confirmed the biological function of *RgLRR29*-OX in *R. glutinosa* under the treatments of root exudates, *F. oxysporum* and comprehensive stress of root exudates and *F. oxysporum*, *RgLRR29*-OX and WT seedlings were cultivated in pots and irrigated with root exudates solution, conidial suspensions of *F. oxysporum*, and a mixture of the two in the root regions. After 15 days of treatment, the transgenic plants displayed significantly less wilting and lower degree of root browning and rot symptoms than the WT seedlings. Under the treatments of root exudates, *F. oxysporum* and comprehensive stress of root exudates and *F. oxysporum*, the antioxidant system including SOD, POD, and CAT in *RgLRR29*-OX also showed higher activities compared with WT seedlings. In contrast, the content of MDA was lower than WT under the treatments of root exudates, *F. oxysporum* and comprehensive stress of root exudates and *F. oxysporum* (**Figure 8D**), and it may positively regulate the immune defense of *R. glutinosa* against replant disease. The results indicated that *RgLRR29*-OX showed higher resistance to root exudates, *F. oxysporum*, and the double stress compared with WT seedling.

## Discussion

Increasing evidence has suggested that replant disease is the results of the comprehensive effects of various factors in the “plant-soil-microbes” system. Among these, the main causes that contribute to the occurrence of replant disease have been found to be allelotoxic substances, microbes, and the imbalance of rhizosphere microecology induced by their interaction (Bertin et al., 2003; Li et al., 2014; Yang RX et al., 2014; Ji et al., 2021; Feng et al., 2022). In other words, allelotoxic substances promote continuously the proliferation of harmful microbes in rhizosphere soils, which then infect the replanted plants and disturb their normal growth (Zhang and Lin, 2009; Dong et al., 2016; Zhang et al., 2016; Westerveld and Shi, 2021). Root exudates and litter mixtures are main sources of allelotoxic substances, which play important roles in regulating microbial community (Jin et al., 2022). Root exudates were be focused on numerous studies of replant disease (Zhang et al., 2013). In this study, we also indicated that root exudates of *R. glutinosa* in replanted condition could induce chemotaxis proliferation of its pathogenic pathogen *F. oxysporum*. It was verified once again that the imbalance of microecological environment in the rhizosphere soils caused by root exudates possibly is an important factor causing *R. glutinosa* replanted disease.

Some studies on the mechanism of replant disease have indicated that the interaction affects each other among allelotoxic substances, harmful microbes and immune-related proteins are closely related to the death of replanted *R. glutinosa* (Wu et al., 2013; Zhang et al., 2013). To reveal the roles of immune-related proteins during the formation of replant disease, this study analyzed in detail the effects of allelotoxic substances using root exudates collected using specific device (Feng et al., 2022) and harmful microbes on *R. glutinosa* seedlings from tissue culture under controlled conditions. Using tissue culture seedlings and controlled conditions could avoid the interference of other biotic or abiotic stress factors in the field. In previous studies of replant disease, single or several representative kinds of allelotoxic substances and rhizosphere microbes were used to simulate the interactions between two factors in rhizosphere soils (Zhang et al., 2015; Li et al., 2016). Root exudates have significant allelopathic activity and are the main source of allelotoxic substances (Liu et al., 2010; Zhang et al., 2015). These results more effectively reflected the interactions between allelotoxic substances and harmful microbes compared to replacing several specific allelotoxic substances by root exudates. Among nine *RgLRRs* in *R. glutinosa*, there were at least eight *RgLRRs* that responded to the comprehensive stress of root exudates and *F. oxysporum*. A total of six *RgLRRs* (*RgLRR19*, *RgLRR21*, *RgLRR25*, *RgLRR26*, *RgLRR27*, and *RgLRR29*) were highly expressed in the early stages and decreased in the later stages under the stresses of root exudates and *F. oxysporum*. It is worth noting that their expression patterns showed similar trends with the expression patterns of *RgLRRs* at different developmental stages of replanted *R. glutinosa* in natural fields (Xie et al., 2019). This result suggested that early infection of *F. oxysporum* in *R. glutinosa* activated the expression of *RgLRRs*, but with the continuous infection triggered by root exudates, the plant immune system was gradually inhibited. In conclusion, these results preliminarily confirmed that the interaction between root exudates and rhizosphere microbes affected the expression of key immune-related proteins in *R. glutinosa* during the formation of replant disease.

Previous studies have demonstrated that the function of plant immune proteins will be gradually diminished during the process of *F. oxysporum* infection of *R. glutinosa* (Chen et al., 2019). In the field, the continuous accumulation of allelochemicals in rhizosphere soils of replanted plants has led to the proliferation of harmful microbes in the rhizosphere (Zhang et al., 2013; Jiao et al., 2015; Dong et al., 2016; Zhou and Wu, 2018; Westerveld and Shi, 2021). During this, the functions of plant immune proteins will be seriously diminished in comparison to responses against single pathogenic microbes, making replanted plants more susceptible to disease infection. LRR-RLKs have been identified in many plant species and have been implicated in regulating the processes of plant growth, development, and responses to biotic and/or abiotic stresses (Jones and Dangl, 2006; de Lorenzo et al., 2009; Halter et al., 2014). Most of the RLKs identified as being involved in plant defense are of the LRR-RLK class including the rice Xa21 protein and the *Arabidopsis* Flagellin Sensitive 2 (FLS2) and bacterial translation elongation factor EF-Tu receptor (EFR) (Song et al., 1995; Chinchilla et al., 2007; Schoonbeek et al., 2015). During the interaction of plants and microbes, overexpression of *LRR-RLK* gene*s* can increase plant resistance to pathogens. For example, overexpression of *OsSERK1* in two rice cultivars led to an increase in host resistance to a blast fungus (Hu et al., 2005). *GbSOBIR1* played a critical role in *Gossypium barbadense* resistance to *Verticillium dahliae* (Zhou et al., 2018). Overexpression of *MdBAK1* in *Malus domestica* inhibited colonization of *F. oxysporum* in host plants (Liu et al., 2022). Therefore, the enhancement of the activity of immune-related proteins might increase the resistance of replanted plants to harmful microbes. In this study, root segments displaying transient overexpression of *RgLRR19*, *RgLRR21*, *RgLRR23*, or *RgLRR29* showed higher resistance levels to a mixed stress of RE and FO. Furthermore, stable overexpression of *RgLRR29* effectively improved resistance of *R. glutinosa* under the treatments of root exudates, *F. oxysporum* and comprehensive stress of root exudates and *F. oxysporum*. There existed a trade-off between the immune responses and plant growth and development, so overexpression of *RgLRR29* affected the root expansion. However, it has been shown that *RgLRR29*-OX could decrease the mortality levels of *R.glutinosa* seedlings compared to WT seedlings. These results suggested that RgLRR proteins may be key proteins in the interactions between root exudates and plant, and that the activity degree of *RgLRRs* determined the damage degree of replanted *R. glutinosa*. In addition, this study preliminarily indicated that activity of *RgLRRs* weakened with the proliferation of *F. oxysporum* induced by root exudates in replanted *R.glutinosa*.

In conclusion, this study confirmed that root exudates could induce *F. oxysporum* to colonize in the rhizosphere soils of *R. glutinosa* and promote the formation of replant disease. RgLRRs played important roles in the process of replant disease. Through the overexpression of key *RgLRR*, we found that the overexpressing *R. glutinosa* seedlings had enhanced resistance to the combined stresses of root exudates and *F. oxysporum*. Hence, this study preliminarily confirmed the interaction among plant immune key proteins, allelopathic substances, and *F. oxysporum*, thereby providing a key breakthrough for further revealing the mechanism of *R. glutinosa* replant disease. In addition, the acquisition and identification of an RgLRR protein has provided a key clue for preventing or reducing the harmful effects of replant disease.

## Data availability statement

The original contributions presented in the study are included in the article/**Supplementary Material**. Further inquiries can be directed to the corresponding author.

## Author contributions

CY and ZX conceived the study. ZZ supervised this research. CY, SQ, ZY, and ZX performed the experiments. ML, LG, JZ and SSQ provided technical help. CY and ZX analyzed the data and wrote the manuscript. All authors contributed to the article and approved the submitted version.

## Funding

This research was financially supported by the National Natural Science Foundation of China (Grant No. 81573538), the Natural Science Foundation of Fujian Province (2020J01531), the Distinguished Youth Fund of Fujian Agriculture and Forestry University (Kxjq20010), and the Special Fund for Science and Technology Innovation of Fujian Agriculture and Forestry University (CXZX2020010A).

## Conflict of interest

The authors declare that the research was conducted in the absence of any commercial or financial relationships that could be construed as a potential conflict of interest.

## Publisher’s note

All claims expressed in this article are solely those of the authors and do not necessarily represent those of their affiliated organizations, or those of the publisher, the editors and the reviewers. Any product that may be evaluated in this article, or claim that may be made by its manufacturer, is not guaranteed or endorsed by the publisher.

## Supplementary material

Supplementary Figure 1 Phenotypic changes of root segments with transient overexpression and interference of different *RgLRRs* under the treatments of root exudates, *F. oxysporum* and comprehensive stress of root exudates and *F. oxysporum*. OX, Overexpression; Ri, RNA interference; CK, Empty vector as control; DAT, Days after treatment; RE, the treatment of root exudates; FO, the treatment of *F. oxysporum*; RE+FO, the comprehensive treatment of root exudates and *F. oxysporum*; There were three replicates for each treatment.

Supplementary Figure 2 Trypan blue staining analysis of root segments with transient overexpression and RNAi of nine *RgLRRs* under the treatments of root exudates, *F. oxysporum* and comprehensive stress of root exudates and *F. oxysporum*. CK, Empty vector as control; RE, the treatment of root exudates; FO, the treatment of *F. oxysporum*; RE+FO, the comprehensive treatment of root exudates and *F. oxysporum*; Bar=5 mm, There were three replicates for each treatment.

Supplementary Figure 3 Antioxygen enzyme activities and MDA content analysis of root segments with transient overexpression and RNAi of nine *RgLRRs* under the treatments of root exudates, *F. oxysporum* and comprehensive stress of root exudates and *F. oxysporum*. OX, Overexpression; Ri, RNA interference; CK, Empty vector as control.
