# Supplementary material for: Functional analysis of Rehmannia glutinosa key LRR-RLKs during interaction of root exudates with Fusarium oxysporum reveals the roles of immune proteins in formation of replant disease
Source: Front Plant Sci. 2022 Oct 31;13:1044070. doi: 10.3389/fpls.2022.1044070 (PMC9660255; doi:10.3389/fpls.2022.1044070)
Supplement: Supplementary file 1 [file Table_1.docx]

**SUPPLEMENTAL MATERIALS**

**Table S1** Primer Datasets Used in This Study

| 1. Primer sequences of overexpression vectors | | |
| --- | --- | --- |
| Gene name | Primer sequences (5’ →3’) | Tm (℃) |
| *RgLRR19* | F: acgggggactcttgaccatggATGGGGACTGTTCGAGTTATTTTT | 60.6 |
|  | R: gggaaattcgagctaggtcaccCTATCGAGCAGTCGGTCGTGC | 63.1 |
| *RgLRR21* | F: tagaggatccccgggggtaccATGGCGGGGCGAGTGGCG | 59.6 |
|  | R: cgatcggggaaattcgagctCTTATAACATGTCACTTGCTTCATGATC | 60 |
| *RgLRR23* | F: tagaggatccccgggggtaccATGCAAAACCCACAAAATTCCA | 62 |
|  | R: cgatcggggaaattcgagctCTTAGCCACCACTTGGCTCGT | 60.6 |
| *RgLRR24* | F: tagaggatccccgggggtaccATGGACTTTCAATTCTTCTCTTTTCTT | 60.5 |
|  | R: cgatcggggaaattcgagctcTCAGAATGCATCCTCAGTAGTAGCA | 61.1 |
| *RgLRR25* | F: tagaggatccccgggggtaccATGGCGGCTCTGAACCTTCT | 61.2 |
|  | R: cgatcggggaaattcgagctctcaGAAAGGAGTAATACTTCCACTTCTC | 60.9 |
| *RgLRR26* | F: tagaggatccccgggggtaccATGGCTGTGATTTTGAAGTGCG | 62.6 |
|  | R: cgatcggggaaattcgagctcCTATGGTGTGACACCAGGAGGTG | 61.6 |
| *RgLRR27* | F: tagaggatccccgggggtaccATGTACAGAAGAAATATGATATATTTTGC | 61.3 |
|  | R: cgatcggggaaattcgagctcCTACGAAGAAAGCGCGAGTTCA | 62.5 |
| *RgLRR29* | F: acgggggactcttgaccatggATGATCATGTCTCTCATTCTCGGC | 62.2 |
|  | R: gggaaattcgagctaggtcaccTCATCTTGGACCAGAAAGCTCC | 60.8 |
| *RgLRR33* | F: tagaggatccccgggggtaccATGGAAAGAGGAAAGTCCCTGC | 61.5 |
|  | R: cgatcggggaaattcgagcTCTTATCTCGGGCCGGAGAGTT | 61.2 |

| 1. Primer sequences of RNAi vectors | | |
| --- | --- | --- |
| Gene name | Primer sequences (5’ →3’） | Tm (℃) |
| *RgLRR19* | F: ACCAGGTCTCAGGAGCCAAGAAGATGGGAAAAGTA | 62.5 |
|  | R: ACCAGGTCTCATCGTAGGAGGATGACACCAAAACT | 63.4 |
| *RgLRR21* | F: ACCAGGTCTCAGGAGCACGTCTGAGACATCCAAAT | 63. 5 |
|  | R: ACCAGGTCTCATCGTTGTAACCGAATGAGCCTATC | 62. 8 |
| *RgLRR23* | F: ACCAGGTCTCAGGAGCAATGAAGCAAACAAGCC | 64.3 |
|  | R: ACCAGGTCTCATCGTTTCTCGTGGAGCCTAAAA | 63.5 |
| *RgLRR24* | F: ACCAGGTCTCAGGAGGTAAATGACACCGCCGTCCT | 63.7 |
|  | R: ACCAGGTCTCATCGTGTAAATGACACCGCCGTCCT | 63.1 |
| *RgLRR25* | F: ACCAGGTCTCAGGAGAACCTACTGCTATCCTCCTG | 62 |
|  | R: ACCAGGTCTCATCGTTGTGGCTCAAATCAATGTC | 63.4 |
| *RgLRR26* | F: GTATGCTTCCAACGAAAACTCGACCATTTTAACGTGTC | 63 |
|  | R: ACCAGGTCTCATCGTTCCTCAGGATGAAGAAAATG | 64.2 |
| *RgLRR27* | F: GTATGCTTCCAACGAAAAGCTGCCTTATGAACTTGG | 65.2 |
|  | R: ACCAGGTCTCATCGTGTATGCTTCCAACGAAAA | 64.5 |
| *RgLRR29* | F: ACCAGGTCTCAGGAGGTCAGGGTTTATCAGGTTCT | 62.2 |
|  | R: ACCAGGTCTCATCGTTGTTGAATGTTTTGGTAGGA | 63.5 |
| *RgLRR33* | F: ACCAGGTCTCAGGAGTGCCCCTGAGTATTTATCG | 63.4 |
|  | R: ACCAGGTCTCATCGTCGTTTTGTGATTGCTCCC | 65.4 |

| 1. Primer sequences of subcellular localization vectors | | | |
| --- | --- | --- | --- |
| Protein name | Primer sequences (5’ →3’) | | Tm (℃) |
| RgLRR19 | F: gacgagctgtacaagctcgagATGGGGACTGTTCGAGTTATTTTT | 60.6 | |
|  | R: gtggtggtggtggtgctcgagctatCGAGCAGTCGGTCGTGC | 58.4 | |
| RgLRR21 | F: gacgagctgtacaagctcgagATGGCGGGGCGAGTGGCG | 61.5 | |
|  | R: gtggtggtggtggtgctcgagttatAACATGTCACTTGCTTCATGATC | 56.8 | |
| RgLRR23 | F: gacgagctgtacaagctcgagATGCAAAACCCACAAAATTCCA | 62 | |
|  | R: gtggtggtggtggtgctcgagtTAGCCACCACTTGGCTCGT | 58.7 | |
| RgLRR24 | F: tagaggatccccgggggtaccATGGACTTTCAATTCTTCTCTTTTCTT | 60.5 | |
|  | R: cgatcggggaaattcgagctcTCAGAATGCATCCTCAGTAGTAGCA | 61.1 | |
| RgLRR25 | F: gacgagctgtacaagctcgagATGGCGGCTCTGAACCTTCT | 61.2 | |
|  | R: gtggtggtggtggtgctcgagTCAGAAAGGAGTAATACTTCCACTTCTC | 60.9 | |
| RgLRR26 | F: tagaggatccccgggggtaccATGGCTGTGATTTTGAAGTGCG | 62.6 | |
|  | R: cgatcggggaaattcgagctccTATGGTGTGACACCAGGAGGTG | 60.3 | |
| RgLRR27 | F: tagaggatccccgggggtaccATGTACAGAAGAAATATGATATATTTT | 57.5 | |
|  | R: cgatcggggaaattcgagctccTACGAAGAAAGCGCGAGTTCA | 61.3 | |
| RgLRR29 | F: acgggggactcttgaccatggATGATCATGTCTCTCATTCTCGGC | 62.2 | |
|  | R: gggaaattcgagctaggtcaccTCATCTTGGACCAGAAAGCTCC | 60.8 | |
| RgLRR33 | F: gacgagctgtacaagctcgagATGGAAAGAGGAAAGTCCCTGC | 61.5 | |
|  | R: gtggtggtggtggtgctcgagtTATCTCGGGCCGGAGAGTT | 59.5 | |

| 1. Primer sequences of qRT-PCR | | | | | |
| --- | --- | --- | --- | --- | --- |
| Gene Name | Primer sequences (5'→3') | | | Tm (℃) | |
| *RehLRR19* | | F: GTCTGAACTGTGGTGGTA | 60 | |  |
|  |  | R: CCTGGTCCTACTAACAACA | 59.9 | |  |
| *RehLRR20* | | F: CAAGGTTCTTCTGTTGTCTC | 60.1 | |  |
|  |  | R: GCGGTAACTGATATGGAATC | 59.9 | |  |
| *RehLRR21* | | F: GGTTCTTCTGTTGTCTCTG | 59.3 | |  |
|  |  | R: CGGTAACTGATATGGAATCG | 59.6 | |  |
| *RehLRR22* | | F: TCTCCACTCTCATCAATCTC | 60 | |  |
|  |  | R: CGGTGAAGTTGTTGTGAG | 60 | |  |
| *RehLRR23* | | F: TATCATCCTCCAAGTGCTC | 60.1 | |  |
|  |  | R: CTCTATGCTCGGTCTATCAT | 59.9 | |  |
| *RehLRR24* | | F: CCGATTGCTCACTATTCAC | 59.9 | |  |
|  |  | R: GGAGTTCACCGTACAGTT | 60.4 | |  |
| *RehLRR25* | | F: CTTCTTCAGATGTGGAGTTG | 59.9 | |  |
|  |  | R: AGGAGGATAGCAGTAGGT | 59.8 | |  |
| *RehLRR26* | | F: TCTCTTCTCCAACTCAACC | 60.2 | |  |
|  |  | R: GCAAATCAATCCCTTCCTC | 60.1 | |  |
| *RehLRR27* | | F: AGAATTGGACAGGCTACC | 60.1 | |  |
|  |  | R: AGGTGCTCATAGAGTGTTC | 60.4 | |  |
| *RehLRR28* | | F: CGGACACAGCAATAACAC | 60.2 | |  |
|  |  | R: CCACCTCTGCCTATTACAT | 60.1 | |  |
| *RehLRR29* | | F: GGATGAAGACTCTGTGGAT | 60 | |  |
|  |  | R: ACCTGATAAACCCTGACTAG | 59.9 | |  |
| *RehLRR30* | | F: CCCGAGTATTATCAGACCTT | 60 | |  |
|  |  | R: CAATGACCAGAGCACCTA | 60.1 | |  |
| *RehLRR31* | | F: CCACCTCTGCCTATTACAT | 60.1 | |  |
|  |  | R: CGGACACAGCAATAACAC | 60.2 | |  |
| *RehLRR32* | | F: TGGACTTGCCGATAGATG | 60.3 | |  |
|  |  | R: GGACGAGGATAACTCTTGT | 60 | |  |
| *RehLRR33* | | F: ATGGTAGTGTGGCATCTAG | 60.1 | |  |
|  |  | R: CGAAGTCATCATCCAACAG | 59.8 | |  |

Tm: melting temperature.
