# Supplementary figures and images for: Functional analysis of Rehmannia glutinosa key LRR-RLKs during interaction of root exudates with Fusarium oxysporum reveals the roles of immune proteins in formation of replant disease
Source: Front Plant Sci. 2022 Oct 31;13:1044070. doi: 10.3389/fpls.2022.1044070 (PMC9660255; doi:10.3389/fpls.2022.1044070)

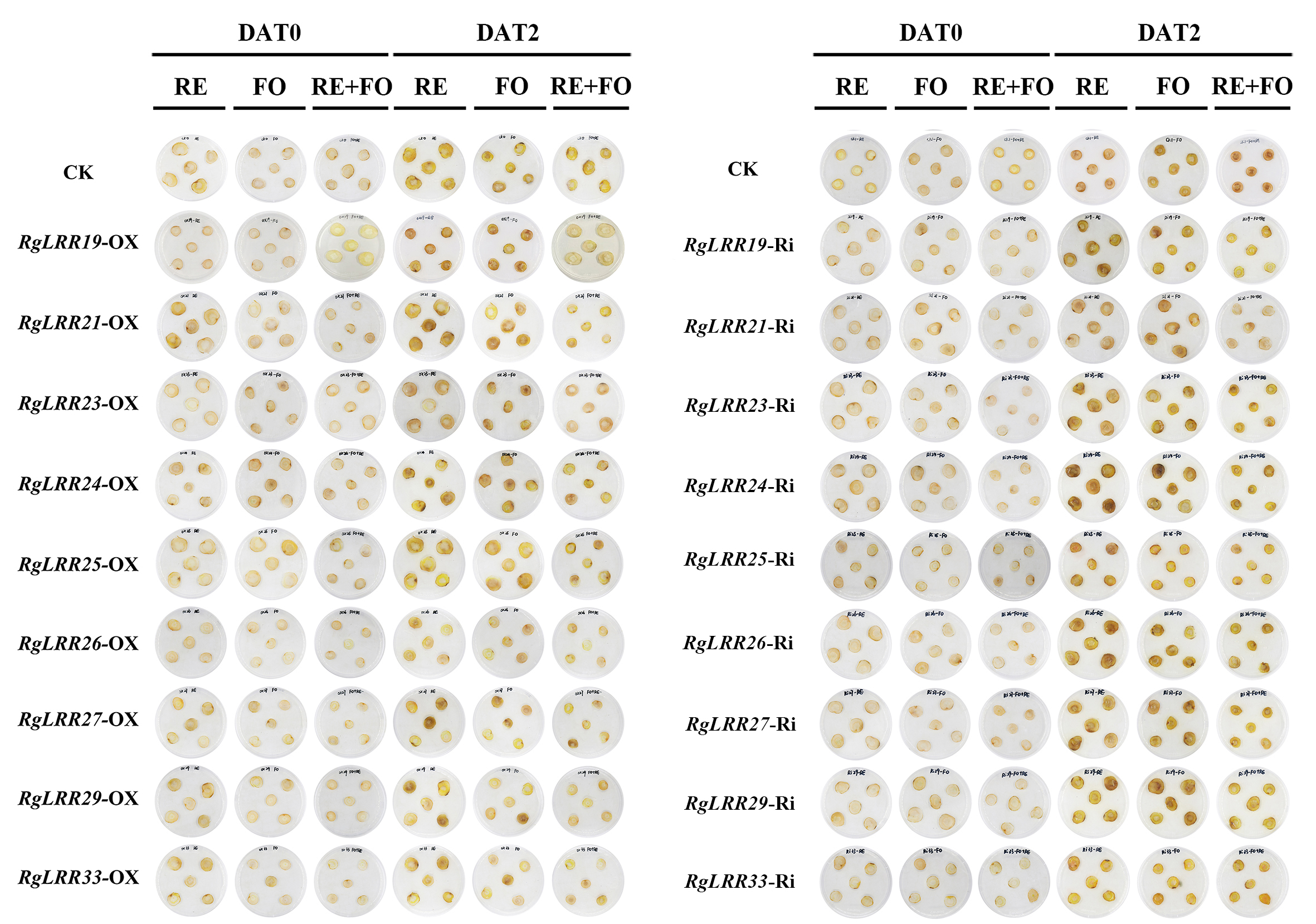

Supplement: Supplementary Figure 1 — Phenotypic changes of root segments with transient overexpression and interference of different RgLRRs under the treatments of root exudates, F. oxysporum and comprehensive stress of root exudates and F. oxysporum. OX, Overexpression; Ri, RNA interference; CK, Empty vector as control; DAT, Days after treatment; RE, the treatment of root exudates; FO, the treatment of F. oxysporum; RE+FO, the comprehensive treatment of root exudates and F. oxysporum; There were three replicates for each treatment. [file Image_1.jpeg]

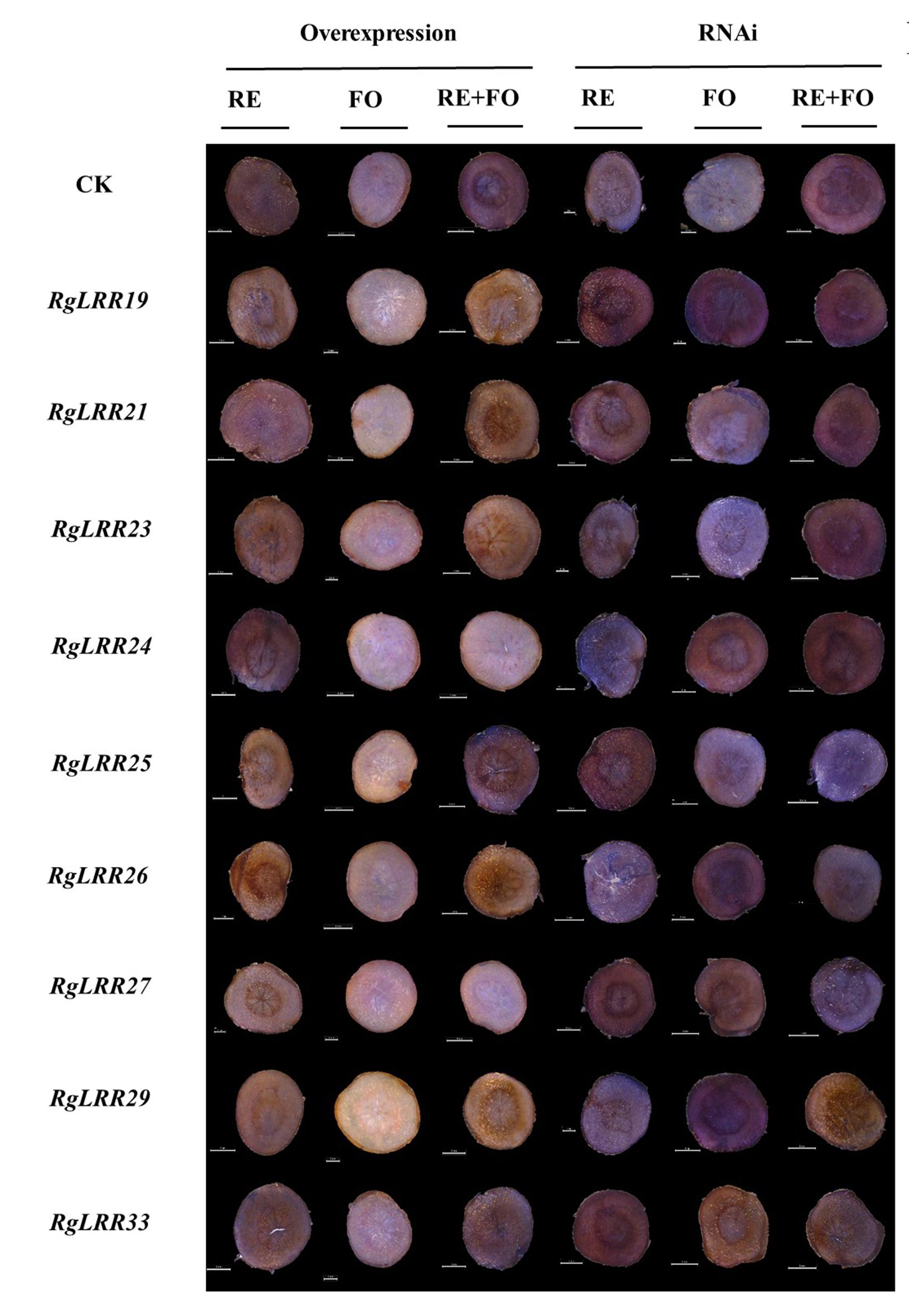

Supplement: Supplementary Figure 2 — Trypan blue staining analysis of root segments with transient overexpression and RNAi of nine RgLRRs under the treatments of root exudates, F. oxysporum and comprehensive stress of root exudates and F. oxysporum. CK, Empty vector as control; RE, the treatment of root exudates; FO, the treatment of F. oxysporum; RE+FO, the comprehensive treatment of root exudates and F. oxysporum; Bar=5 mm, There were three replicates for each treatment. [file Image_2.jpeg]

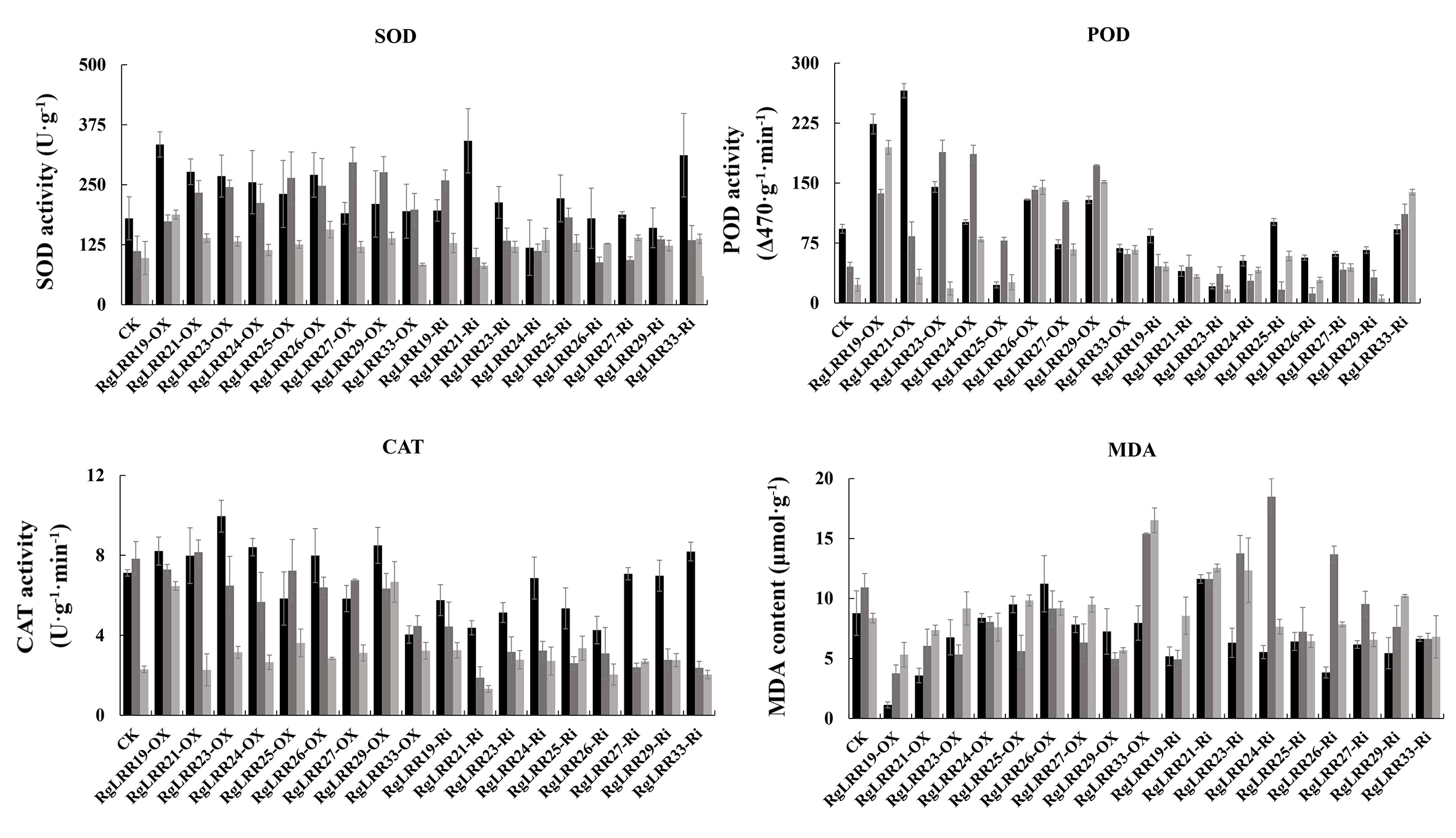

Supplement: Supplementary Figure 3 — Antioxygen enzyme activities and MDA content analysis of root segments with transient overexpression and RNAi of nine RgLRRs under the treatments of root exudates, F. oxysporum and comprehensive stress of root exudates and F. oxysporum. OX, Overexpression; Ri, RNA interference; CK, Empty vector as control. [file Image_3.jpeg]
